# Highly sensitive blood-based biomarkers detection of beta-amyloid and phosphorylated-tau181 for Alzheimer’s disease

**DOI:** 10.3389/fneur.2024.1445479

**Published:** 2024-09-02

**Authors:** Wei Yang, Fulin Guan, Lihui Yang, Guangli Shou, Fangfang Zhu, Yuanyuan Xu, Ying Meng, Min Li, Wanli Dong

**Affiliations:** ^1^Department of Neurology, First Affiliated Hospital of Soochow University, Suzhou, China; ^2^Department of Neurology, Second Affiliated Hospital of Bengbu Medical College, Bengbu, China; ^3^Department of Neurology, Suzhou Dushu Lake Hospital, Suzhou, China

**Keywords:** blood biomarker, Alzheimer’s disease, mild cognitive impairment, plasma p-tau181, Aβ40, Aβ42

## Abstract

**Background:**

Plasma biomarker has the potential to be the reliable and propagable approach in the early stage diagnosis of Alzheimer’s disease (AD). However, conventional methods appear powerless in the detection of these biomarkers at low concentrations in plasma. Here, we determined plasma biomarker concentrations of patients across the AD spectrum by an improved digital enzyme-linked immunosorbent assay (ELISA) technique. Confirms the predictive and diagnostic value of this method for AD patients and study the relationships between these biomarkers and cognitive status.

**Methods:**

Plasma concentrations of amyloid-beta 40 (Aβ40), amyloid-beta 42 (Aβ42) and plasma phosphorylated tau at threonine 181 (p-tau181) were determined in 43 AD patients, 33 mild cognitive impairment (MCI) patients and 40 normal cognition (NC) subjects as healthy controls using the improved digital ELISA technique. In addition, all subjects were required to receive neuropsychological assessments.

**Results:**

Plasma p-tau181 level showed certain discrepancies between NC and MCI (*p <* 0.05), AD (*p <* 0.01) groups. The level of plasma Aβ42 (*p <* 0.05) and Aβ40 (*p <* 0.01) was significantly different between AD and NC group. The p-tau181 level was able to distinguish AD (AUC = 0.8768) and MCI (AUC = 0.7932) from NC with higher accuracy than Aβ42/Aβ40 ratio (AUC = 0.8343, AUC = 0.6569). Both p-tau181 (CDR: *r* = 0.388 *p* < 0.001; MMSE: *r* = −0.394 *p* < 0.001) and Aβ42/Aβ40 ratio (CDR: *r* = −0.413 *p* < 0.001; MMSE: *r* = 0.358 *p* < 0.001) showed stronger positive correlation with clinical dementia rating (CDR) and mini mental state examination (MMSE) scores than Aβ42 (CDR: *r* = −0.280 *p* = 0.003; MMSE: *r* = 0.266 *p* = 0.005) or Aβ40 (CDR: *r* = 0.373 *p* < 0.001; MMSE: *r* = −0.288 *p* = 0.002) alone.

**Conclusion:**

Plasma p-tau181 level and Aβ42/Aβ40 ratio showed promising values in diagnosis of AD and MCI. Our results indicate that this improved digital ELISA diagnosis approach can facilitate early recognition and management of AD and pre-AD patients.

## Introduction

Alzheimer’s disease (AD) is the most prevalent cause of dementia among the elderly. The 2021 World Alzheimer Report indicates that over 55 million individuals worldwide are currently suffering from dementia. However, it is estimated that 75% of these cases remain undiagnosed, with this figure potentially rising to 90% in developing countries (1). Recent estimates indicate that the number of individuals aged 60 years or older with AD dementia in China is over 9.83 million (2). The symptoms of AD become progressively worse over time, with brain damage occurring 20 years or more before clinical symptoms appear (3–10). Individuals with MCI do not yet meet the criteria for dementia, but have a greater possibility of developing into this condition (11, 12). MCI is thought to be closely related to the risk of incident dementia, whether due to AD or even earlier, such as subjective cognitive decline (SCD) (13, 14). Therefore, early diagnosis allows for early intervention and treatment trials.

Previous diagnoses of AD mainly rely on cognitive performance, neuropsychological assessment and biomarkers detected in the cerebrospinal fluid (CSF) or through amyloid positron emission tomography (PET) (15). However, the diagnostic latency, invasiveness, expense and dependence on associated infrastructure of those approaches limit their promotion in clinical practice. In contrast, blood biomarkers have a promising value in clinical practice due to their cost-effectiveness, non-invasiveness, and easy accessibility (16). The assessment of blood biomarkers facilitates the early identification of individuals at risk of developing AD, thus representing a pivotal step toward effectively tackling this urgent public health concern (17). In recent years, some ultra-sensitive measurement of low-abundance biomarkers have been gradually applied to the study of AD protein markers, with sensitivity improving by up to 1,000-fold over conventional ELISA (18–20), leading to the availability of detecting AD-relevant biomarkers in blood samples (21). Researchers have examined the core AD biomarkers based on the “A/T/N” framework in a Han Chinese cohort (22). Despite advancements in quantifying plasma biomarkers like amyloid beta (Aβ1-42 and Aβ1-40) and phosphorylated tau (pTau), technical challenges persist due to the varying costs and usage complexities of detection methods, which necessitate further refinement for extensive clinical adoption (23). Standardization is another critical issue; the absence of uniform procedures in blood sample management, from collection to analysis and reporting, could compromise the reliability of biomarker measurements and impede their application in clinical and research realms (24). The quest for accuracy and robustness in diagnosing Alzheimer’s disease (AD) using blood biomarkers is fraught with challenges, particularly when dealing with diverse community populations. The interference of confounding factors, both systemic and biological, further complicates the accurate detection of these biomarkers, highlighting the need for more sophisticated analytical techniques to isolate their effects. Early diagnosis of AD is also a significant hurdle, as the subtle pathological changes in the initial stages of the disease are tough to discern without highly sensitive detection methods. Capturing the minute variations in blood biomarkers is equally challenging and demands technology that is not only sensitive but also precise (25). Moreover, the journey from research to clinical practice involves a meticulous implementation roadmap that encompasses various stages, including study design, sample handling, biomarker assessment, and reporting of findings. This transition is a gradual process that is still in progress. Lastly, while blood biomarkers offer the benefits of being less invasive and more cost-effective, it is imperative to weigh these advantages against the overall impact on patients and the healthcare system in practical scenarios (23). In summary, the detection of blood biomarkers still faces multiple challenges in achieving clinical application, and further research and technological development are needed to overcome these issues.

The immunoassay used here is an improved digital ELISA. The achievement of signal amplification through dividing of samples into hundreds of micro-reactors and further sealing the reactor which allow the subsequent enzyme-catalyzed reactions happens in the sealed environment, which enables the detection of low abundance protein in blood. It is characterized by accuracy, efficiency, cost-effectiveness, and ease of operation and holds promise in tackling the current challenges associated with biomarkers. Digital ELISA technology is widely used in clinical simultaneous detection of multiple cytokines (26). For instance, the concentrations of interleukin-4 (IL-4) and IL-6 in the peripheral blood of children with asthma were found to be significantly elevated in comparison to healthy controls, whereas the concentrations of interferon-γ (IFN-γ) were significantly decreased (27). The serum levels of IL-6, IL-8, IL-17 and other cytokines are altered in patients with breast cancer (28, 29). Patients with arthritis have abnormal serum cytokine concentrations, such as tumor necrosis factor-α (TNF-α), IL-1, and IL-17, compared with healthy individuals (30, 31).

Here, we applied the improved digital ELISA technique to detect the levels of Aβ40, Aβ42, p-tau181 at low concentrations in individuals with or without cognitive impairment, in according with the first aim of this study, which was to verify the feasibility of the improved digital ELISA technique for the detection of peripheral biomarkers in pre-AD and AD patients. Subsequently, the associations between peripheral biomarkers and different stages of cognitive function were analyzed, as well as the potential utility of plasma biomarkers to diagnose AD.

## Methods

### Study participants and neuropsychological tests

All participants underwent a comprehensive cognitive status and neuropsychological assessment, including the Mini Mental State Examination (MMSE), Clinical Dementia Rating (CDR), the Activity of Daily Living Scale (ADL), and the Hachinski Ischemic Scale. All patients were required to have an insider to provide an evaluation of their functional abilities. The CDR score of normal subjects was 0, while those diagnosed with MCI exhibited a CDR of 0.5 and an MMSE score ranging from 20 to 24 (32). AD patients have a CDR value of 1+. The clinical diagnosis of probable MCI or AD was based on the National Institute on Aging–Alzheimer’s Association (NIA-AA) guidelines (2018, 33).

It is essential to obtain comprehensive information regarding the participants’ demographics, medical history and family history. Meanwhile all MCI and AD patients underwent a battery of tests, including a complete blood count, blood glucose, blood electrolytes, blood urea nitrogen, serology for syphilis, thyroid function, and CT or MRI scans to exclude other potential causes of their dementia.

Informed consent was provided from all volunteers or their legal guardians. The medical ethics committee of Bengbu Medical College approved protocols for this study [2023 (276)].

### Inclusion and exclusion criteria

43 AD and 33 MCI patients were recruited over the period from July 2022 to October 2023 from the Department of Neurology in the Second Affiliated Hospital of Bengbu Medical College and the Fourth Affiliated Hospital of Soochow University.

The inclusion criteria were that all the participants must be over 40 years of age, diagnosis of MCI or AD. All the participants were required to have a reliable insider who could assist in the completion of the clinical visits as needed. A total of 40 normal cognition (NC) over 40 years old from the hospital physical examination center, were recruited as control participants, absence of neuropsychiatric disease, stroke, dementia, and underlying diseases.

The exclusion criteria were subjects with any concomitant neurological, psychiatric or significant medical illness known to affect cognitive function including Parkinson’s disease, Huntington disease, seizure disorder, multiple sclerosis, cerebrovascular disease and brain tumor, or history of major depression, anxiety, or other mental diseases, which makes patient unable to accomplish the assessment of cognitive impairment.

### Sample preparation

Peripheral venous blood samples were collected into EDTA tubes via standard procedures. The collected blood sample was centrifuged for 10 min at 3000 rpm. Subsequently, plasma supernatant was aliquoted into polypropylene tubes and stored at −80°C until further use.

### Materials and operational procedure

Materials, including capture beads, streptavidin-β-galactosidase (SβG), detection antibodies, sample diluent, buffer-1/2, microfluidic chip, fluorogenic substrate and sealing oil were purchased from ColorTech (Suzhou) Biotechnology company (Suzhou, China).

Plasma samples were removed from refrigerator and placed at room temperature for 30 min. Samples were then diluted 4x using sample diluent.

**Figure 1:**
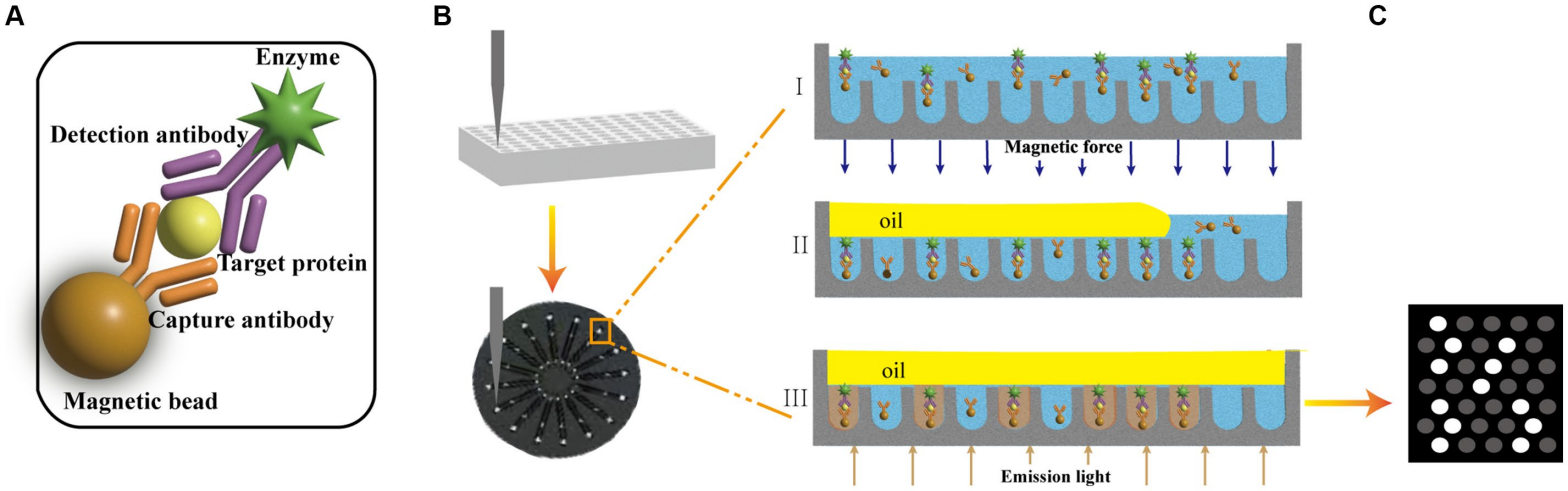
**(A)** Encoded magnetic beads are combined with antigens and enzyme labels to form a sandwich structure complex. **(B)** The plasma mixed with magnetic beads was injected into the flow channel of the disc with 16 integrated microchannels. The coded microbeads automatically enter the chip with the liquid to be analyzed. The magnet helps the magnetic microbeads enter the holes and increases the filling rate (I). The microbeads enter the holes and the enzymes on the beads surface hydrolyze the fluorogenic substrate. A fluorocarbon oil is applied to seal the beads inside different microwells. The beads that are not inside chambers are washed out (II); Light is applied to excite the coding microspheres and liquids in the microwells (III). **(C)** Images of fluorescent molecules are captured dynamically.

The 10 μL of capture beads suspensions, 15 μL of the detection antibody solution and 150 μL sample (or calibrator) were mixed and incubated at room temperature for 30 min (60 min for p-tau181). The beads were separated and washed three times with 200 μL of buffer-1. After washing, 70.0 μL SβG solution was added and incubated for 5 min (10 min for p-tau181). A sandwich structure was formed by magnetic beads, capture antibody, antigen, detection antibody and labeled enzyme (Figure 1A). The beads were separated and washed seven times with 200 μL of buffer-1, and once with 200 μL of buffer-2. Then the beads were resuspended in 20 μL of buffer-2 for further detection.

The beads dispersion was mixed with the fluorogenic substrate and then loaded into the microfluidic chip that has an array of microreactors. The magnet helps the magnetic microbeads enter the holes and increases the filling rate (Figure 1B-I). The size of each microreactor is designed to ensure only one bead can enter. Then the sealing oil was used to seal the microreactors and remove the beads that did not fall into microreactors (Figure 1B-II).

Enzymes on the beads surface will hydrolyze the fluorogenic substrate in the microreactor and the fluorescent products will accumulate with time (Figure 1B-III). The signal will be read and analyzed (Figure 1C). The concentration of the target sample can be obtained by comparing the signal for unknown samples to the calibration curve.

### Statistical analysis

The extreme values of each plasma biomarker, defined as those at least three times the standard deviation (SD) of the mean, were excluded. Normally distributed continuous variables were expressed as mean ± SD, while the skewed distributed continuous variables were described by the median and quartile 1 (Q1) to quartile 3 (Q3). Number (*n*) and percentages (%) were employed for categorical variables. Categorical variables were tested using Pearson chi-square tests. One-way analyses of variance (ANOVA) with the Kruskal-Wallis test was employed for the comparison of continuous variables with unequal variance. *Post hoc* pairwise comparisons was used to evaluate differences among multi-groups and adjusted significance by Bonferroni. Categorical variables were compared using the Pearson’s chi-squared test.

Scatter plots were used to illustrate the distributions of original plasma biomarkers’ levels of the different groups. The associations between CDR, MMSE scores and plasma biomarkers were assessed using partial correlation analyses with the adjustment for age, sex, and education year as covariates. Education was categorized according to its completion within a six-year timeframe.

*p*-values <0.05 was considered statistically significant. All statistical analyses were analyzed using IBM SPSS Statistics for Windows, version 27.0. The scatter plots and receiver-operating characteristic (ROC) curves were visualized by GraphPad Prism version10.1.2 for Windows with control of age, sex, and education year.

## Results

### Characteristics of participants

A total of 116 participants were recruited, including 40 individuals with normal cognition (NC), 33 with a clinically diagnosis of MCI, and 43 patients with AD patients, as characterized by the CDR score. The demographic data of the study participants were presented in Table 1. We did not find a apparent discrepancy in sex among the three groups. The AD group exhibited a significant difference in age, education years compared to the MCI group. The NC group did not differ significantly in age from the MCI group, nor in education from the AD group. As expected, the AD group exhibited the lowest level of education. Following Bonferroni correction, MMSE and CDR scores were found to be significantly different among the three groups.

**Table 1:** Demographic characteristics of subjects.

	Total (*n* = 116)	NC (*n* = 40)	MCI (*n* = 33)	AD (*n* = 43)
Gender, M/F	39/77 (50.6%)	18/22 (81.2%)	12/21 (57.1%)	9/34 (26.5%)
Age, years, Mean (SD)	63.8 (12.7)	59.4 (11.7)^c^	58.2 (12.8)^c^	72.3 (8.6)^a, b^
Education, years, ≤ 6/>6	73/43 (169.7%)	27/13 (207.7%)^b^	14/19 (73.7%)^a, c^	32/11 (290.9%)^b^
MMSE, Median [Q1, Q3]	23.0 [16.0, 28.0]	29.0 [27.0, 30.0]^b, c^	23.0 [20.0, 25.0]^a, c^	13.0 [9.0,17.0]^a, b^
CDR, Median [Q1, Q3]	0.5 [0, 1.0]	0 ± 0^b, c^	0.5 [0.5, 0.5]^a, c^	1.0 [1.0, 2.0]^a, b^

NC, normal cognition; MCI, mild cognitive impairment; AD, Alzheimer’s disease; CDR, Clinical Dementia Rating Scale. SD, standard deviation; MMSE, mini-mental state examination; Q1, quartile 1; Q3, quartile 3; ^a^Significant values versus NC (normal cognition). ^b^Significant values versus MCI (mild cognitive impairment). ^c^Significant values versus AD (Alzheimer’s disease).

### AD biomarkers in plasma across groups

**Figure 2:**
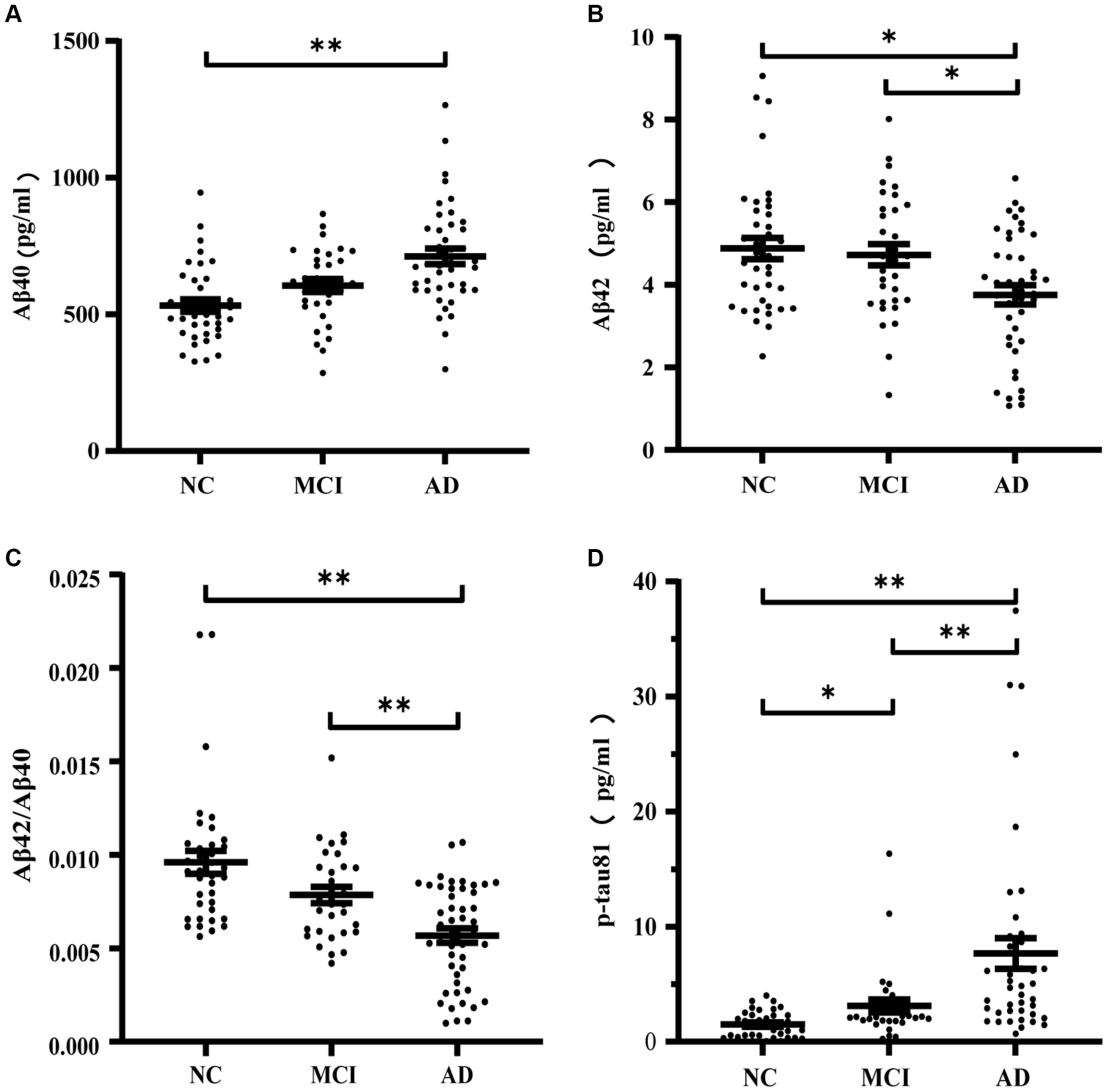
Concentrations of Aβ40 **(A)**, Aβ42 **(B)**, Aβ42/Aβ40 **(C)**, p-tau181 **(D)** in normal cognition subjects, MCI and AD patients. *p ≤ 0.05 and **p ≤ 0.01.

Table 2 and Figure 2 illustrate the levels of plasma biomarkers detected in the different cognitive performance groups. Aβ40 demonstrated significant differences only between participants with NC and AD (Table 2; Figure 2A). No significant discrepancy of Aβ40 was observed between participants with MCI and other groups. Aβ42 and Aβ42/Aβ40 ratio exhibited a descending trend, with the lowest values observed in the AD groups compared to the NC and MCI groups (Table 2; Figures 2B,C). With regard to Aβ42 and Aβ42/Aβ40 ratio, no significant difference was found between participants with NC and MCI. Conversely, plasma p-tau181 exhibited an upward trend across groups, with significant differences observed among the three participant groups (Table 2; Figure 2D).

**Table 2:** Plasma biomarker concentrations and ratios of subjects.

	Total (*n* = 116)	NC (*n* = 40)	MCI (*n* = 33)	AD (*n* = 43)
Aβ40 (pg/ml), Median [Q1, Q3]	609.98 [493.42, 726.15]	496.19 [430.92, 626.11]^c^	620.42 [533.79, 709.83]	680.35 [589.08, 817.20]^a^
Aβ42 (pg/ml), Median [Q1, Q3]	4.27 [3.44, 5.48]	4.62 [3.47, 5.82]^c^	4.69 [3.60, 5.88]^c^	3.95 [2.61, 5.14]^a, b^
Aβ42/Aβ40, Median [Q1, Q3]	0.0074 [0.0056, 0.0093]	0.0091 [0.0068, 0.0106]^c^	0.0074 [0.0059, 0.0093]^c^	0.0058 [0.0035, 0.0080]^a, b^
p-tau181 (pg/ml), Median [Q1, Q3]	2.27 [1.49, 4.17]	1.41 [0.46, 2.30]^b, c^	2.16 [1.81, 3.26]^a, c^	4.76 [2.49, 8.78]^a, b^

NC, normal cognition; MCI, mild cognitive impairment; AD, Alzheimer’s disease; CDR, Clinical Dementia Rating Scale; SD, standard deviation; MMSE, mini-mental state examination; Q1, quartile 1; Q3, quartile 3; Aβ, amyloid-beta protein; t-tau, total tau; NfL, neurofilament proteinlight chain; p-tau181, tau phosphorylated at threonine 181. ^a^Significant values versus NC (normal cognition). ^b^Significant values versus MCI (mild cognitive impairment). ^c^Significant values versus AD (Alzheimer’s disease).

### Associations between plasma biomarkers and cognition

**Figure 3:**
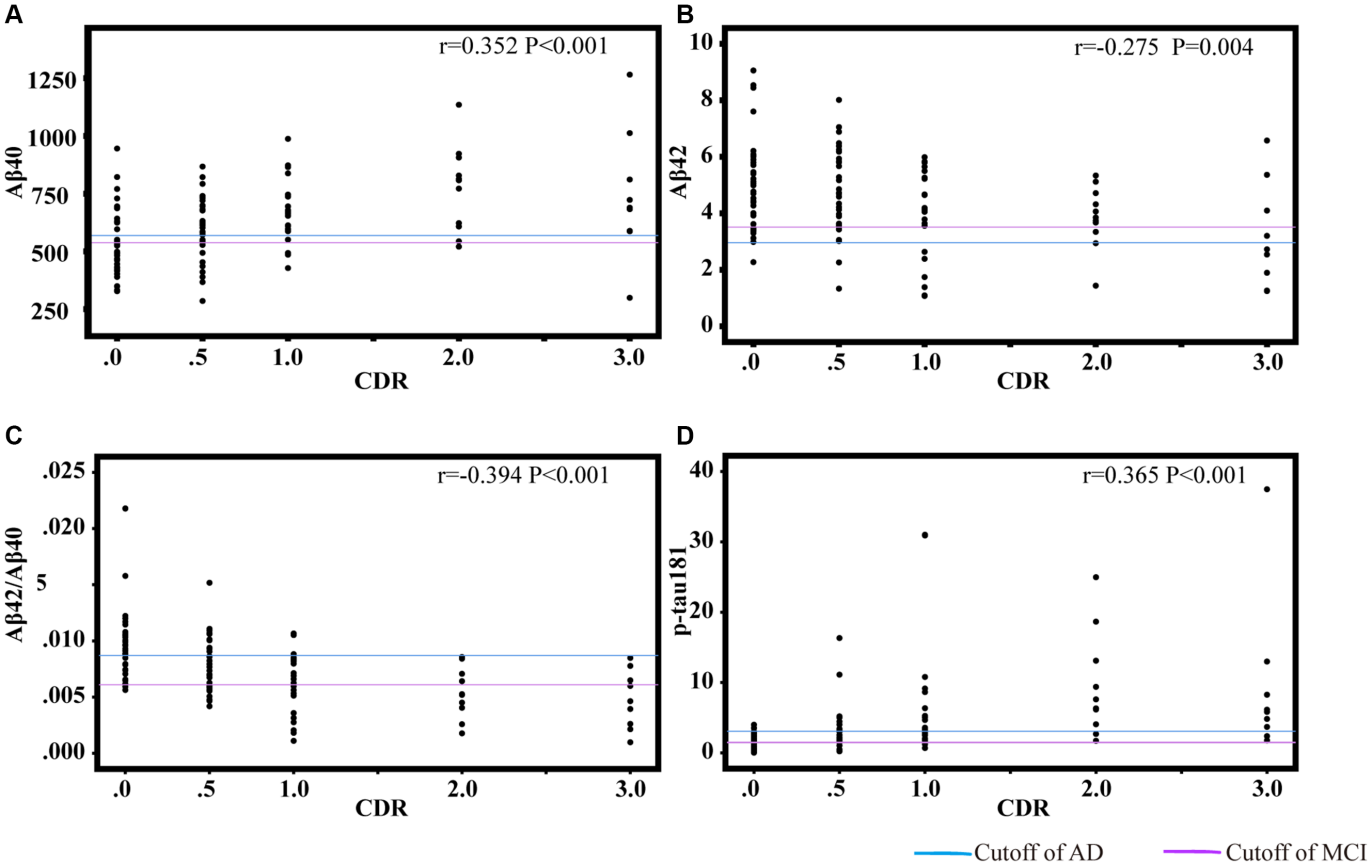
Scatter plots of CDR and plasma biomarkers of Aβ40 **(A)**, Aβ42 **(B)**, Aβ42/Aβ40 **(C)**, p-tau181 **(D)**. The partial correlation coefficients (*r*) were adjusted for age, gender, and education year. *p* < 0.05 was considered statistically significant after using multiple comparisons by Bonferroni correction. CDR, clinical dementia rating; Aβ, amyloid-beta protein; p-tau181, tau phosphorylated at threonine 181.

**Figure 4:**
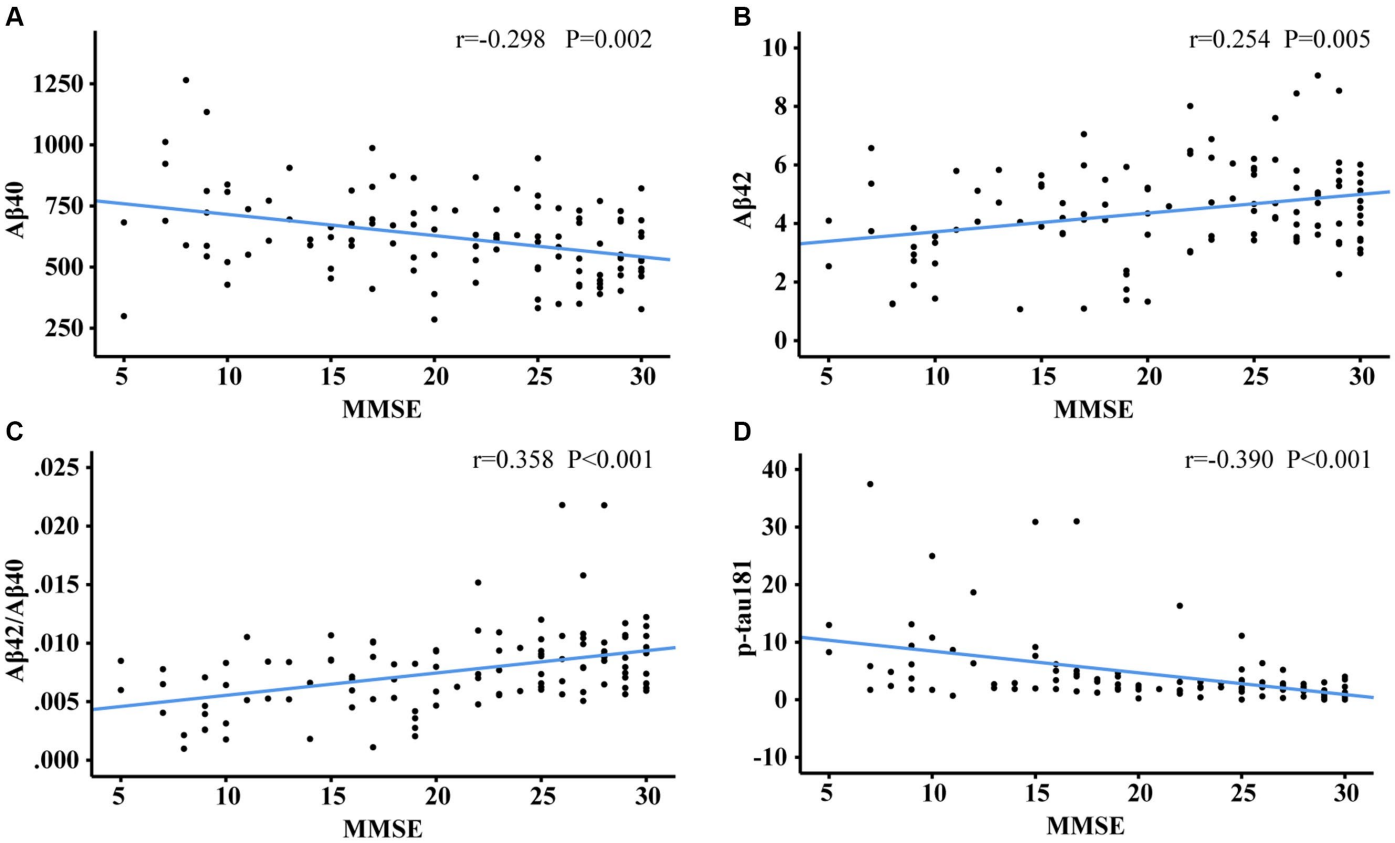
Scatter plots of MMSE and plasma biomarkers of Aβ40 **(A)**, Aβ42 **(B)**, Aβ42/Aβ40 **(C)**, p-tau181 **(D)**. The partial correlation coefficients (*r*) were adjusted for age, gender, and education year. *p* < 0.05 was considered statistically significant after using multiple comparisons by Bonferroni correction. MMSE, mini-mental state examination; Aβ, amyloid-beta protein; p-tau181, tau phosphorylated at threonine 181.

Figures 3, 4 showed the partial correlation matrix between three plasma biomarkers and CDR and MMSE scores after adjusting age, sex and education years.

In Figure 3, Aβ40 showed a positive correlation with CDR (*r* = 0.352, df = 108, *p* < 0.001). Aβ42 (*r* = −0.275, df = 2, *p* = 0.004) and Aβ42/Aβ40 ratio (*r* = −0.394, df = 107, *p* < 0.001) had an inverse correlation with CDR. P-tau181 demonstrated a positive correlation with CDR (*r* = 0.365, df = 105, *p* < 0.001). As shown in Figures 3C,D, the cut-off values of Aβ42/Aβ40 ratio and p-tau181 were able to discriminate individuals with cognitive impairment from normal controls. The performance improves with increasing CDR. As demonstrated in Figure 4, Aβ42/Aβ40 ratio (*r* = 0.358, df = 107, *p* < 0.001) and p-tau181 (*r* = −0.394 df = 105, *p* < 0.001) also showed a stronger correlation with MMSE than Aβ42 (*r* = 0.266, df = 108, *p* = 0.005) or Aβ40 (*r* = −0.288, df = 108, *p* = 0.002). Higher Aβ40 and p-tau181 were correlated with worse cognitive scores, which correspond to lower Aβ42.

**Figure 5:**
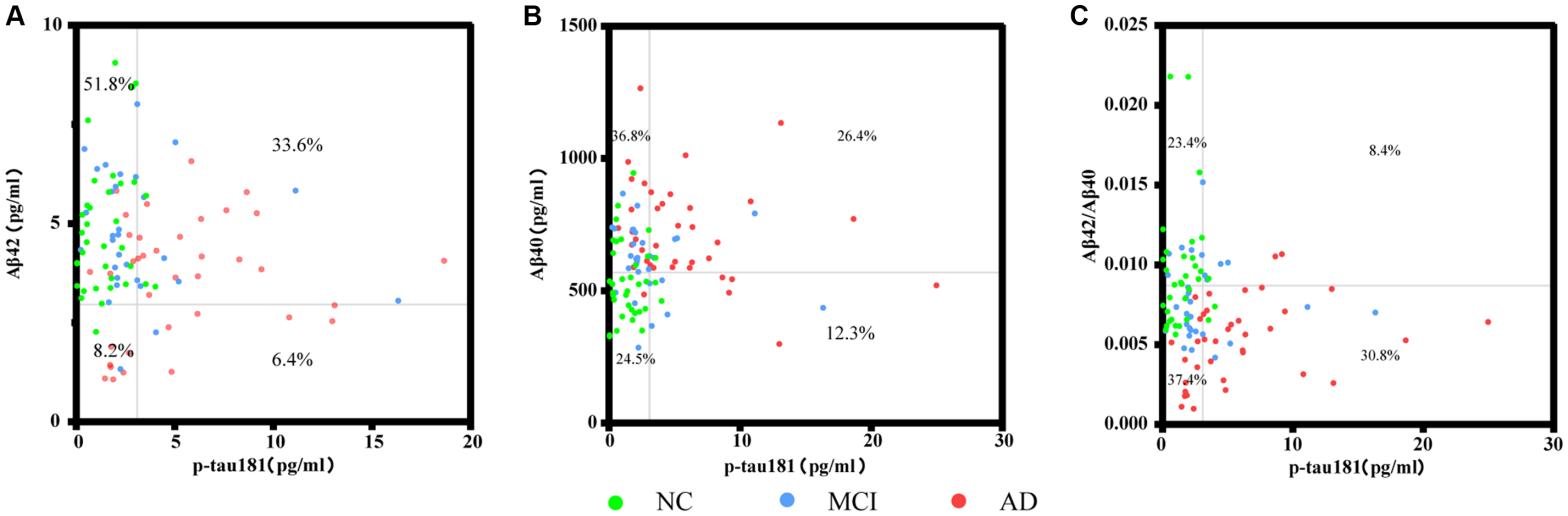
Scatter plots represent the correlation between plasma biomarkers in each pairwise analysis among the different groups. Each point refers to the value of indicated measures of a single participant, and the grey lines indicate the cutoff for each biomarker. Percentages indicate the proportions in each quadrant. Four extreme values were not shown in panel **(A)**, and three points in panels **(B,C)** separately, but they were included in the statistical analyses.

Figure 5 shows the correlation between plasma biomarkers in each pairwise analysis, respectively. Participants were distributed to four quadrants according to the respective cutoff value for plasma biomarkers of AD. From the distribution in Figure 5A, it can be seen that Aβ42 performed well in distinguishing controls, but not AD subjects. As illustrated in Figures 5B,C, individuals with AD were predominantly located in the double-positive quadrant. P-tau 181 and Aβ42/Aβ40 ratio exhibited the highest concordance (30.8%) in differentiating positive individuals from negative individuals, with 63.4% of AD patients being distinguished.

### The value of plasma biomarkers in predicting cognitive status

**Figure 6:**
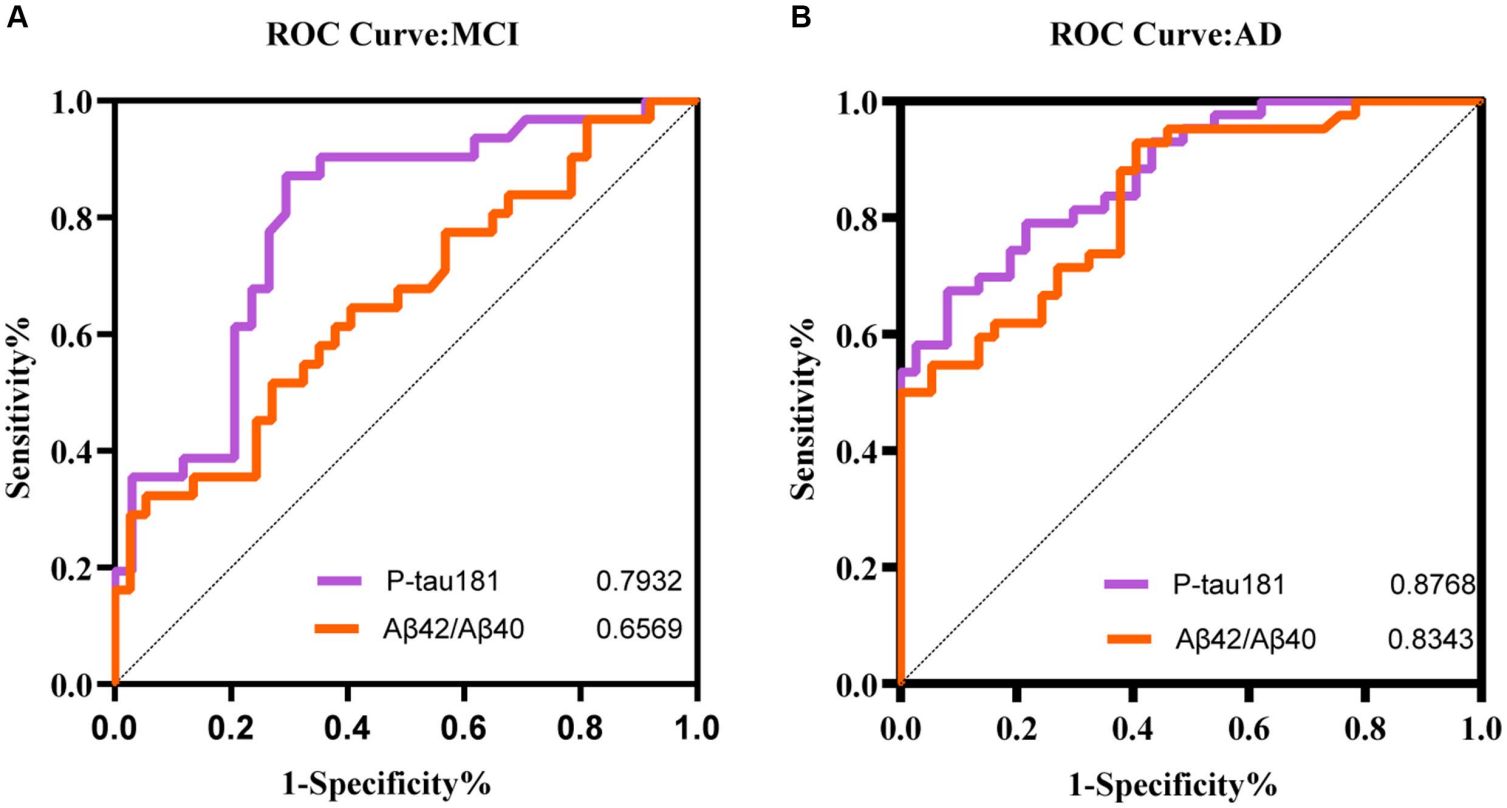
ROC curve analyses of different models for MCI **(A)** and AD **(B)** patients.

To figure out if plasma biomarkers are sufficient to identify MCI and AD from non-AD, we performed receiver operator characteristic (ROC) analysis on the diagnostic accuracy of p-tau 181 and the Aβ42/Aβ40 ratio. This analysis demonstrated a higher correlation with cognitive scores. The results demonstrated that p-tau 181 (AUC = 0.8768) and Aβ42/Aβ40 ratio (AUC = 0.8343) were able to classify the AD groups with higher accuracy than MCI (AUC = 0.7932 and 0.6569, respectively) (Figure 6). The performance of p-tau 181 was marginally superior to that of the Aβ42/Aβ40 ratio.

## Discussion

The principal findings of the present study were as follows: (1) Plasma Aβ42, and the Aβ42/Aβ40 ratio exhibited a declining trend, whereas plasma Aβ40 and p-tau181 demonstrated an upward trajectory in conjunction with the aggravation of cognitive impairment. (2) Both plasma p-tau181 and the Aβ42/Aβ40 ratio were valuable markers for the diagnosis of AD. P-tau181 was found to be a more effective indicator of clinical cognitive performance. (3) The digital ELISA was identified as a promising and reliable approach for clinical screening of patients with MCI or AD. It has the characteristics of high efficiency and low cost, which would enable early diagnosis and treatment at earlier phases of research, potentially accelerating the discovery of new biomarkers for complex diseases, such as neurological disorders.

Aβ accumulation and hyperphosphorylated tau protein have been considered as potential triggers and/or drivers in the development of AD (33). Plasma concentrations of Aβ42 and the Aβ42/Aβ40 ratio are significantly reduced in AD, indicating the presence of Aβ deposition in the brain. Some have proposed that the Aβ42/Aβ40 ratio offers superior predictive accuracy in determining Aβ status compared to Aβ42 alone (34, 35). Plasma p-tau 181 was inversely changed in AD, which is likely indicative of the presence of neurofibrillary tangles within the brain (36). This study also demonstrated that p-tau 181 and the Aβ42/Aβ40 ratio were tightly correlated with the CDR and MMSE scores, in agreement with previous studies (37, 38). Generally, the p-tau181 biomarker demonstrated the highest sensitivity and specificity in discriminating between control subjects and patients diagnosed with Alzheimer’s disease (AD). These results are highly consistent with prior studies. The biomarkers Aβ42, Aβ40, total tau (t-tau) and p-tau181 showed good diagnostic performance (39–41). Furthermore, some studies have confirmed the high predictive value of plasma p-tau in diagnosing AD (16, 37, 42). Nevertheless, the correlations we observed between CDR, MMSE and plasma Aβ42 were weaker even compared with Aβ40 and showed low accuracy for diagnosis of AD. The suboptimal predictive accuracy of plasma Aβ42 may be attributed to the limited sensitivity of this improved digital ELISA technique in quantifying the overall levels of plasma Aβ. Further strengthening improvement of the reagent and procedure is necessary to enhance their effectiveness.

Traditional ELISA is known for its ease of use, cost efficiency, and versatility but is limited by longer processing times, particularly with multiple washing steps, and lower sensitivity. It can also be prone to interferences that may result in false positives or negatives (43). Single Molecule Arrays (SiMoA) offer high sensitivity by detecting individual proteins but require specific complex equipment and techniques (44). The paper-based lateral flow immunoassay (dLFI) provides a rapid, visual, and practical point-of-care method for detecting AD biomarkers within 30 min, similar to ELISA results, but without the need for specialized equipment. However, it may not match the sensitivity and specificity of laboratory-level ELISA and might not detect all relevant AD biomarkers (45). The immunomagnetic exosomal polymerase chain reaction (iMEP) is a highly sensitive technique for the rapid detection of amyloid-beta and phosphorylated tau proteins in blood exosomes, essential for AD diagnosis. It allows for precise detection and simultaneous analysis of multiple biomarkers but may be limited by its technical complexity and the need for potentially costly specialized equipment and reagents (46). The colorimetric and surface-enhanced Raman scattering (SERS) dual-mode magnetic immunosensor combines colorimetric and SERS techniques for high-sensitivity detection of AD biomarkers, with rapid and intuitive visual color change results. It is particularly adept at identifying low concentrations of p-tau396, 404, aiding in early diagnosis. However, it requires specific SERS equipment, expertise, and may be costly (47, 48). Lastly, the densely aligned carbon nanotube sensor array is a highly sensitive and accurate platform for detecting AD biomarkers at femtomolar concentrations with multiplex detection capabilities. Despite these strengths, its cost and the need for specialized equipment and expertise may limit broader accessibility (49). This was the first study to use the improved digital ELISA to detect of blood biomarkers for AD, which achieves signal amplification by quantifying of single molecules. Digital ELISA represents the latest breakthrough in protein detection, specifically targeting proteins present at minimal concentrations (50–52). Such a platform is urgently needed to unlock the potential biomarker, which is rapidly trending toward low abundance biomarkers associated with disease states.

In our study, we used an external magnetic field to enhance the loading efficiency of the magnetic beads. The low cost of our novel system greatly increases its potential for commercialization. The primary approach to improve the digital ELISA involves evaluating the variation in fluorescence intensity of the liquid in the microwell, which can be used to determine the concentration of the target protein. The traditional digital technique restricts the magnetic bead signal to binary values of 0 or 1, whereas our methodology encompasses a wider range of nuances. Access to its unique ability to quantify single molecules facilitates a more comprehensive understanding of the biological aspects associated with disease progression or the impact of treatments during initial stages of investigation, potentially accelerating the identification of novel biomarkers for complex conditions such as neurodegenerative diseases, facilitating the identification and diagnosis of neurological conditions. The implementation of this proactive strategy will empower individuals at risk to proactively engage in preventive measures, effectively delaying the development of, disease. This progress signifies a notable step toward prompt early intervention and improved clinical management, ultimately delivering tangible benefits to both researchers and patients.

There were several limitations to our study. First and foremost, the sample size was small. This small sample size was partly due to the Corona Virus Disease 2019 (COVID-19) epidemic, but mainly because the rigorous exclusion criteria for participants. The reliability of cytokines is a important issue that can be greatly influenced by confounding variables such as concurrent medical conditions and the use of other medications. In the case of COVID-19 infection, infected blood samples were being available. The stringent selection procedure enabled us to achieve a high level of reliability in our analyses. Secondly, it was not possible to determine whether plasma biomarkers (Aβ40, Aβ42, and P-tau181) correlated with corresponding CSF biomarkers of AD, as CSF collection was rarely accepted by both patients and controls. Further research with larger sample sizes is necessary to validate our findings. Thirdly, the individuals involved in our research were sourced from two different backgrounds, which inevitably led to some inequality. Age, gender and year of education were adjusted for in the statistical models. Fourthly, learning effects (53, 54) and intrusion errors are inevitable in tests evaluating comparable cognitive domains, which may result in potential fluctuations in assessing the cognitive performance of the study subjects. Fifthly, the diagnosis of AD was primarily based on clinical standards rather than pathological evidence from CSF or amyloid/tau PET scans. The lack of a gold standard has impeded us from classifying the ‘ATN’ framework (55). Finally, the pre-sample processing of the improved digital ELISA used in this work is artificially based. There is an urgent need to make this improved digital ELISA technique fully automate in order to minimize the operational errors. We are also developing the technology’s multiplex detection capabilities to enable the simultaneous detection of multiple AD core biomarkers.

## Conclusion

In conclusion, we detected Aβ42, Aβ40 and p-tau181 levels in the plasma of AD, MCI and control groups with a high degree of accuracy using this improved digital ELISA technique. Therefore, this technique has the potential to expedite the identification of individuals at risk of dementia, thus contributing to the advancement of AD early screening and clinical drug development for Alzheimer’s disease. Of note, multi-centre, longitudinal and more holistic studies are necessary to verify this methodology and to further substantiate the correlation between plasma biomarkers and cognitive manifestations.

## Data Availability

The raw data supporting the conclusions of this article will be made available by the authors, without undue reservation.
